# Do time trade-off values fully capture attitudes that are relevant to health-related choices?

**DOI:** 10.1007/s10198-018-1017-8

**Published:** 2018-12-31

**Authors:** Anne Spencer, Ewan Tomeny, Ruben E. Mujica-Mota, Angela Robinson, Judith Covey, Jose Luis Pinto-Prades

**Affiliations:** 1 Health Economics Group, College of Medicine and Health, University of Exeter, Exeter, EX1 2LU UK; 2 Liverpool School of Tropical Medicine, Pembroke Place, Liverpool, L3 5QA UK; 3 Norwich Medical School, University of East Anglia, Earlham Road, Norwich, NR4 7TJ UK; 4 Department of Psychology, Durham University, Stockton Road, Durham, DH1 3LE UK; 5 Department of Economics, Universidad de Navarra, 31009 Pamplona, Spain

**Keywords:** Time trade-off method, Utility, Attitudes, Preference elicitation, TTO, I10

## Abstract

Previous research has shown that demographics, beliefs, and self-reported own health influence TTO values. Our hypothesis is that attitudes towards length and quality of life influence TTO values, but should no longer affect a set of related choices that are based on respondents’ own TTO scores. A representative sample of 1339 respondents was asked their level of agreement to four statements relating to the importance of quality and length of life. Respondents then went on to value 4 EQ-5D 5L states using an online interactive survey and a related set of 6 pairwise health-related choice questions, set up, so that respondents should be indifferent between choice options. We explored the impact of attitudes using regression analysis for TTO values and a logit model for choices. TTO values were correlated with the attitudes and were found to have a residual impact on the choices. In particular, those respondents who preferred quality of life over length of life gave less weight to the differences in years and more weight to differences in quality of life in these choice. We conclude that although the TTO responses reflect attitudes, these attitudes continue to affect health-related choices.

**Electronic supplementary material:**

The online version of this article (10.1007/s10198-018-1017-8) contains supplementary material, which is available to authorized users.

## Introduction

The time trade-off (TTO) method is a widely applied method used in health economics to elicit respondent preferences for health state valuation [1] and more recently has been used to estimate the monetary values for health gains [2]. It is well recognised that health state values are influenced by demographic characteristics, such as household income, sex, and level of education [3–5]. A growing literature is also beginning to show the impact that beliefs have upon TTO values.

In an early survey [6], responders’ comments highlight the importance of domains such as satisfaction with life, happiness, and religious beliefs. Though the impact of religion on TTO values has been found to be ambiguous, linked to both lower [7] and higher TTO values [8], in other domains, clear trends have emerged. Augestad et al. [9] investigated whether TTO values were influenced by attitudes towards euthanasia and found that an increase in agreement with the practice of euthanasia resulted in more willingness to trade time and lower TTO values elicited for health states. In another related study, van Nooten et al. [10] investigated the effect of respondents’ subjective life expectancy on their willingness to trade time and found those who believed that they had longer to live were less willing to trade away years and this increased TTO values. More recently, van Nooten et al. [11, 12] found that respondents were more willing to trade life years when they: supported euthanasia, were willing to take more health risks (measured on the Health-Risk Attitude Scale) or had a lower expectation regarding mental ageing. Respondents were less willing to trade life years if they expressed a fear of death or had other important life events taking place within the TTO timeframe [11]. In their discussion, van Nooten et al. [11] highlight the need to check if such relationships hold when the TTO is conducted over a longer duration.

In this paper, we explore the extent to which the trade-off valuations fully capture attitudes that are relevant to health-related choices. Our hypothesis is that attitudes towards length and quality of life influence TTO values. We then go on to test whether these attitudes towards length and quality of life have any residual impact on a set of related choices that are based on respondents’ own TTO scores.

## Methods

### Data

We analysed data from an internet-based survey conducted in June 2014 with a representative UK sample aged 18–70 [13]. The final data set used here contained 1339 respondents. All data collection was managed by *Cint* and was approved by Ethics Committee at Glasgow Caledonian University.

### Survey

The survey was split into four main sections. Prior to valuing any states, in section 1, respondents were asked their age and gender, their own health measured by EQ-5D 5L and questions to assess attitudes. Section 2 had respondents valuing states by TTO, while section 3 asked the respondents direct choices between two lives set up, so that respondents should be indifferent between the two lives offered based on their previous TTO responses. The final section asked respondents demographic questions including employment, marital status, and educational level.

The health states used in the survey were based on the EQ-5D 5L descriptive system. Respondents were randomised into valuing a set of ‘mild’ or ‘moderate’ health states. The health states in the mild group were 11121, 21211, 12212, and 13122 and in the moderate group were 13122, 13224, 23242, and 23314. The states were chosen to have varying degrees of severity on different dimensions while minimising the likelihood that any state would be rated as worse than dead to circumvent the need to value worse than dead. Health state 13122 was common to both groups. Finally, the states were chosen such that there existed strict dominance between at least two states, allowing straight-forward checks for response consistency. We used 20 years throughout the TTO questions as life expectancy of most of the subjects in the survey was at least 20 years. Furthermore, longer TTO durations have been found to produce TTO values consistent with those derived over a 10 year period, and it is only shorter TTO durations that produce most differences [14], so we felt that the choice of the 20 year duration was unlikely to undermine the generalisability of our results.

### TTO questions

**Fig. 1:**
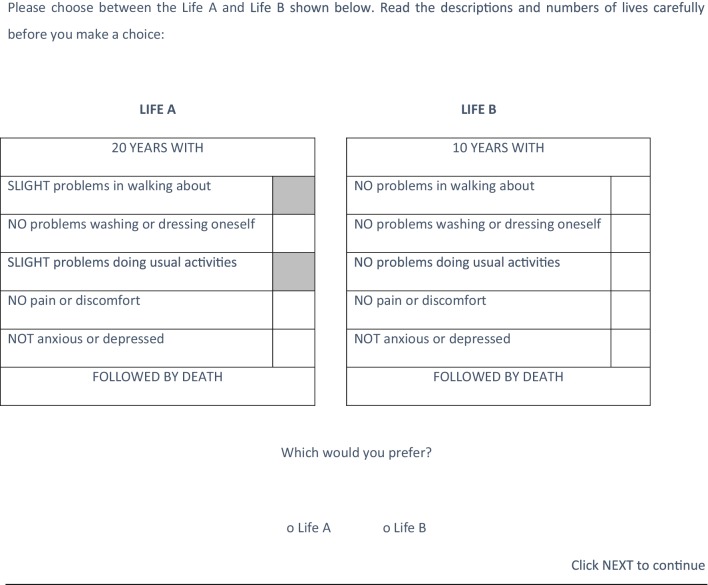
TTO question using 21211 as an example

The TTO questions involved a between-group 2 × 2 design in which respondent were randomised to different TTO variants. The first broad difference between the TTO variants was whether the elicitation procedure was iterative or non-iterative. Respondents were first presented with a choice between 20 years in Life A and 10 years in Life B, as illustrated in Fig. 1 for state 21211. In the iterative procedure, the subsequent choices ‘honed into’ the point of indifference by adjusting the time in full-health in successive 2-year intervals based on the respondent’s previous answers. For example, if the respondent preferred 10 years in Life B to 20 years in Life A, they were then presented with a choice between 8 years in Life B and 20 years in Life (A). If the respondent preferred 20 years in Life A to 10 years in Life B, they were then presented with a choice between 20 years in Life A and 12 years in Life (B). This iterative process continued until they ‘switched’ to preferring Life A to Life B in successive 2 year intervals—or vice versa—they were then asked about the year in between. In the non-iterative procedure, the computer randomly generated the subsequent TTO choices for each health state, and therefore, the choices were not based on a respondent’s previous responses.

The second broad difference between the TTO variants was whether the elicitation procedure for each health state was sequential or concurrent. In sequential procedure, the elicitation procedure was completed for each health state in turn before moving onto value the next heath state. In the concurrent procedure, the elicitation procedures were ‘spliced’ together, so the respondent considered the iteration procedure for the one health state and then the next iteration procedure for the next health state and so on until all four health states were asked, and then, the process was repeated. The health states were valued in the order: 12212, 11121, 13122, and 21211 in the mild group and 23242, 13122, 23314, and 13224 in the moderate group except for the non-iterative concurrent group, where health states were valued in a random order.

For values in which the respondent continually refused to trade, 19, 19.5, and 19.75 years in full-health were offered, to increase sensitivity at this end of the scale. Conversely, for those who continually choose full-health and preferred 1 year in full-health over 20 years in the other health state, they were asked if they would prefer death, and if so these observations were assigned a value of 0 and dropped from the main analysis.

We ran a regression to explore the extent to which we could pool responses from the TTO variants to allow us to focus upon the impact of attitudes towards length and quality of life. The regression model that was constructed contained dummy variables representing the TTO variants and all seven states, with a dummy for the severity of the health states to which the respondent was randomised (mild and moderate). Also included were interaction terms (cross products) between states and TTO variants. We used an *F* test to determine whether the dummies and their interactions were simultaneously zero. This is similar to testing for significant differences between a model with these variant variables added and a model without them, i.e., the difference between full model and reduced models.

### Choice questions

Following the TTO exercises, respondents were presented with six pairwise choices which asked them to directly compare *X* years in one health state and *Y* years in the other. The choice questions were set up in order that they should be indifferent between the two lives offered based on their previous responses. The assumptions required for these choices to hold is mutual utility independence and constant proportional trade-off [15] and would allow for possibility that respondents may discount future life years in their TTO responses.

**Fig. 2:**
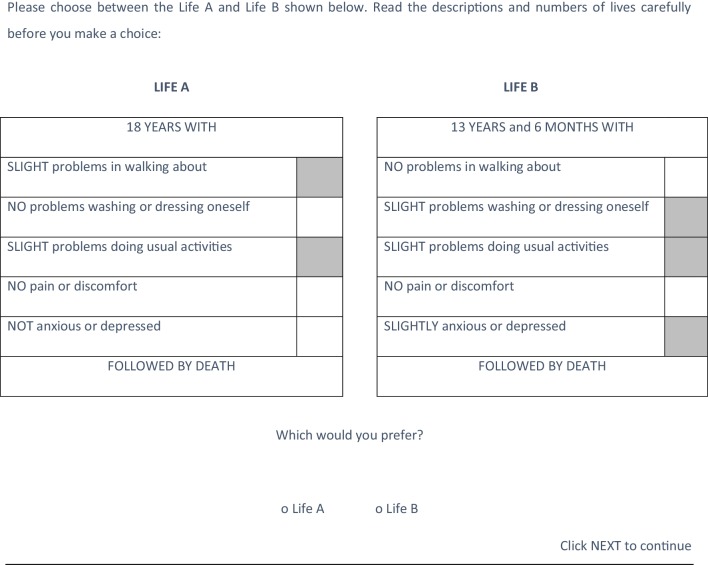
Choice question using 21211 and 12212 as an example

To set up the choice, the computer program would first select the state with the lower utility value. For example, suppose that *U*1 and *U*2 are the TTO utility values for health states 1 and 2, respectively, and that *U*1 $\prec ~$*U*2. The choice questions would present respondents with *X* years in health state 1 and *U*1/*U*2 × *X* years in health state 2. In each choice, one of the two states always appeared in Life A—while the other appeared in Life B—and this was set in advance. The health states included in Life A v Life B comparison were as follows: 11121 vs 21211, 11121 vs 12212, 11121 vs 13122, 21211 vs 12212, 21211 vs 13122, and 12212 vs 13122. Thus, either Life A or Life B could involve the greater number of life years—depending on the respondent’s valuation of the health states in the TTO. The number of years was randomly chosen as either 17, 18, or 19. Therefore, for example, if *U*1 and *U*2 equalled 0.6 and 0.8, respectively, and 18 years was selected as the value of *X, Y* would then be set at 0.6/0.8 × 18 = 13.5 years. Figure 2 illustrates the choice question when if *U*1 = 21211 = 0.6 and *U*2 = 12212 = 0.8, and 18 years was selected as the value of *X*,

### Attitude questions

**Fig. 3:**
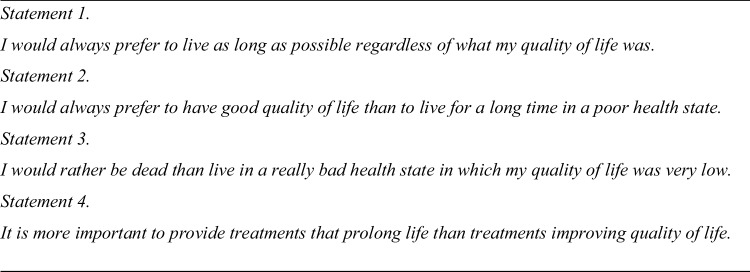
Attitudinal statements presented to respondents

Respondents were asked to state their level of agreement with four statements, as shown in Fig. 3 concerning the relative importance of quantity and quality of life. Responses adopted a five-point Likert scale.

The Cronbach alpha coefficient was used to explore the internal consistency of responses to the Likert scales statements to establish if they were measuring the same underlying construct. In addition, it was important that each scale contributed some unique information to avoid duplication. The Cronbach alpha coefficient ranges from 0 to 1 with low levels indicating lack of internal consistency and very high levels indicating a potential redundancy of one or more scale. The threshold criterion used to determine whether the scales could be summed can vary by the number of items in the scale and whether the analysis is exploratory in nature to devise a new scale. In our analysis, we follow the standard criterion that a Cronbach alpha between 0.70 and 0.90 [16] indicates good internal consistency for exploratory studies and that the scales can be combined [17].

### Exploring the impact of attitudes on TTO values and choices

We explored the impact of the attitude scale using a generalised estimating equation model for TTO values and a logit model for choices which allowed for clustering of responses by respondent. Our hypothesis was that attitudes would affect TTO values, but should no longer affect a set of related choices that are based on respondents’ own TTO scores.

We first estimated a base model that adjusted for demographic variables and included age, age^2^, gender, marital status (dummy for married), age left full-time education and employment status (dummy for employed full time), and the severity of the health states. A secondary analysis estimated the same base model but without the health state severity, to be comparable with the base model used by van Nooten and colleagues [11]. In the event that a variable was found to be insignificant, it was determined whether the variable might be dropped by means of an *F* test (comparing the model with the variable included against a model with them excluded). All regressions and data analyses were handled in STATA Version 14.0 (StataCorp LP, TX, USA).

To establish if and how attitudes affected preferences elicited by TTO, the base model was run with a measure of attitudes towards length and quality of life. For example, suppose that respondents’ attitudes towards length and quality of life could be measured by the variable ATLQL_stan_, derived from one or more attitude statements and that the ATLQL_stan_ score was highest for those who indicated quality of life was most important and lowest for those who considered length more important:

1 $${\text{util}}=\alpha +{\beta _1}{\text{ATLQ}}{{\text{L}}_{{\text{stan}}}}+\{ {\text{BASEMODEL}}\} +\epsilon .$$

If ATLQL_stan_ was then included in the base model, as shown in Eq. (1), a significant negative coefficient would then be expected, indicating those with a preference for quality had a higher willingness to trade-off years, resulting in lower util values.

In the choice questions, Life A or Life B could involve a greater number of life years in a lower quality of life—depending on the respondent’s valuation of the health states in the TTO. Therefore, to explore the impact of attitudes on a respondent’s propensity to choose the life with more life years and lower quality of life, we added a variable years_*δ*_ (Eq. 2) to represent differences in life years between Life A and Life B, so we could distinguish clearly when more life years were preferred:

2 $${\text{year}}{{\text{s}}_\delta }={t_{\text{A}}} - {t_{\text{B}}},$$

where years_*δ*_ ≥ 0 ⇒ *t*_A_ ≥ *t*_B_.

In the base logistic model, characteristics were added as main effects, including severity of the health states (mild vs moderate), along with their interaction with years. The model was subsequently reduced until only pairs with significant interactions remained (verified by *F* tests). To establish if attitudes affected choices, the base model was run with ATLQL_stan_ added (Eq. 3):

3 $${\text{Latent}}\;{\text{propensity}}\;{\text{Life A}}=\alpha +{\beta _1}{\text{ATLQ}}{{\text{L}}_{{\text{stan}}}}+{\text{ }}{\beta _2}{\text{year}}{{\text{s}}_\delta } \times {\text{ATLQ}}{{\text{L}}_{{\text{stan}}}}+\{ {\text{BASEMODEL}}\} +\epsilon .$$

From Eq. (3), we can estimate the probability of choosing Life A using Eq. (4):

4 $$P\left( {{\text{Life}}\;{\text{A}}} \right)=\frac{1}{{1+\frac{1}{{{{\exp }^{({\text{Eq}}.\;(3))}}}}}}.$$

The constant term in Eq. (3) is the latent propensity of choosing Life A for those respondents that valued the health states the same in the TTO study in the mild group and for whom the numbers of life years were the same in Life A and Life B. The variable ATLQL_stan_ explores the extent to which attitudes have a residual impact for these choices that essentially involve two different health states and no differences in length of life. For these choices, we anticipate that all respondents can perceive differences in quality of life and attach importance to these differences given that there are no differences in length of life. Hence, we anticipate the coefficient on ATLQL_stan_ to be non-significant. Of interest to the current study is the interaction term between ATLQL_stan_ and years_*δ*_ (i.e., *β*_2_). Our hypothesis is that if attitudes have a residual impact on choices then for those respondents who have a relative preference for quality of life they will choose the life with the health state which brought them higher utility, and give less weight to the differences in years. For this reason, we expect the coefficient on the interaction term between ATLQL_stan_ and years_*δ*_ (i.e., *β*_2_) to be negative.

## Results

### Descriptive statistics of the respondents and attitudes

In total, there were 1462 respondents who took part in the web-based survey. Prior to analysis exclusions were made as shown in Online Appendix 1 which resulted in a ‘cleaned’ data set of size 1339. The mean respondent age was 45 with a standard deviation of 14 years, and a gender (%) split of 54 female/46 male. Approximately half of the sample were married (49%) and a further 12% lived with a domestic partner. A demographic breakdown of respondents is given in Table 1 and respondents were broadly representative of the general population and web-based surveys of this type. The sample was relatively healthy, with 32% reporting full-health (11111) and 67% no worse than level 2 in any dimension.

**Table 1:** Descriptive statistics

Attributes	Pre-exclusions (*N* = 1462) *N* (%)	Post-exclusions (*N* = 1339) *N* (%)	General population^a^ (%)
Age (years)			
18–19	50 (2.5)	33 (2.5)	–
20–29	288 (14.3)	195 (14.6)	(13.6)
30–39	440 (21.8)	277 (20.7)	(13.2)
40–49	448 (22.2)	302 (22.6)	(14.6)
50–59	443 (22.0)	307 (22.9)	(12.1)
60–69	348 (17.3)	225 (16.8)	(10.8)
Gender			
Male	945 (46.9)	618 (46.2)	(49.2)
Female	1072 (53.1)	721 (53.9)	(50.8)
Economic activity			
Employed full time	856 (42.4)	564 (42.1)	(48.2)
Employed part time	401 (19.9)	277 (20.7)	(13.7)
Retired/can’t work/disabled	353 (17.5)	222 (16.6)	(18)
Student/at school	93 (4.6)	64 (4.8)	(9.2)
Not working/looking for work	148 (7.3)	99 (7.4)	(6.6)
Housewife/househusband	166 (8.2)	113 (8.4)	(4.3)
Marital status			
Married	1009 (50.0)	660 (49.3)	(46.6)
Never married (single)	515 (25.5)	351 (26.2)	(34.6)
Divorced	156 (7.7)	105 (7.8)	(9.0)
Widowed	41 (2.0)	32 (2.4)	(7.0)
Domestic partner	249 (12.4)	159 (11.9)	–
Other/prefer not to state	47 (2.3)	32 (2.4)	(2.8)
Highest level of education			
Junior school	59 (3.0)	38 (2.8)	(23)
Secondary school	580 (29.0)	387 (28.9)	(28)
College/higher education	465 (23.1)	308 (23.0)	(12)
Tech college/teacher training	170 (8.43)	108 (8.1)	(10)
University/open university	744 (36.9)	498 (37.2)	(27)

^a^Taken from the 2011 Census, England and Wales (Fields matched where possible)

**Fig. 4:**
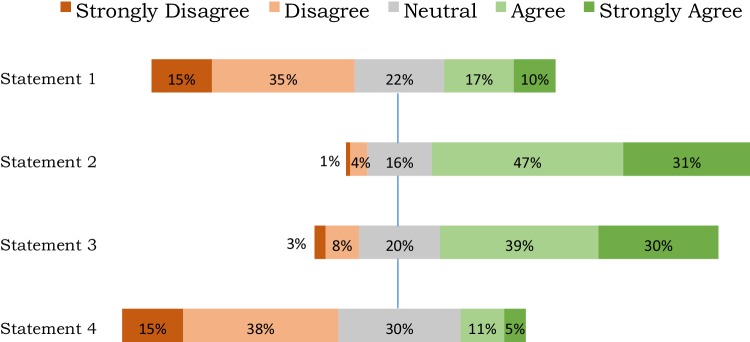
Responses to the attitudinal questions

The responses to the attitudinal questions are shown in Fig. 4. Responses suggested that the majority would be prepared to live a shorter life if spent in good health, with 80% agreeing or strongly agreeing with the second statement: *I would always prefer to have good quality of life than to live for a long time in a poor health state*. The statement which caused the most to be unsure was statement 4 that was asked from a societal perspective, with 53% expressing a view that quality is more important than length. There was a mild trend with age and attitudes, with older people tending to have a preference for longer life.

Table 2 summarises the internal consistency of responses to the attitude statements to establish if they were measuring the same underlying construct. The sign column depicts whether the statements were found to be inversely correlated (depicted by a negative sign), and where the scale was reversed before combining with the other statements. The item-test correlation depicts the correlations of the statements with a summary measure based on all statements and these correlations are all very similar as would be expected if the statements were measuring a similar construct. The item-rest correlations depict the correlation between a statement and a scale formed by all the other statements and ranged from 0.43 to 0.55, providing evidence of adequate item coherence, but not excessive multicollinearity. The average inter-item correlation is the average correlation of the statement with the other statements and correlations are 0.34 or above. The last column shows the Cronbach alpha value if a statement was dropped and showed that the value does not increase if any of the statements were omitted. Finally, the Cronbach alpha coefficient for the combined four statements was 0.708, exceeding the proposed threshold criterion (0.7–0.9) allowing us to combine the four statements into one scale.

**Table 2:** Cronbach’s alpha measure of internal consistency

Item	Sign	Item-test correlation	Item-rest correlation	Average inter-item correlation	Alpha if statement dropped
Statement 1	+	0.767	0.551	0.341	0.608
Statement 2	−	0.685	0.425	0.421	0.685
Statement 3	−	0.762	0.544	0.345	0.613
Statement 4	+	0.705	0.456	0.401	0.667
Test scale^a^				0.377	0.708

^a^Test scale = mean(standardised items); number of observations = 1339 for each item

The four-item scale was active across all values in its range.

A single scale was created by reversing the responses to statements 2 and 3 (as they were inversely correlated with statements 1 and 4) and the four scores summed. This scale was then normalised and standardised (see Online Appendix 2) to create the variable, ATLQL_stan_, that is used throughout the rest of the analyses. The variable ATLQL_stan_ score is highest for those who indicated quality of life is most important and lowest for those who considered length more important.

### TTO variants

We tested the extent to which we could pool TTO values from across the different TTO variants by running a regression model with dummies for the different TTO variants, and used an *F* test to determine whether the dummies and their interactions were simultaneously zero. The regression was run on the cleaned data set of 1339 and the detailed results are shown in Online Appendix 3. The regression results show that we can pool across all TTO variants provided we dropped health state 12212 in the iterative sequential variant and 23314 in the non-iterative sequential variant. The reported results for the remaining analyses are based on the pooled data set.

### The impact of attitudes on TTO values and choice

The base model included demographic variables and health states, and a secondary analysis included only demographic variables. We found qualitatively similar results between the two types of models, so report here the model that included demographic variables and health states. With all variables included, gender and employment were not significant and these variables could be omitted without loss of power^1^. This process established our base model as that shown in Table 3 for the TTO values.

**Table 3:** TTO values

	Base TTO model				The effect of attitudes on TTO values			
util	Coefficient	Std. err.	*z*	*p* > |*z*|	Coefficient	Std. err.	*z*	*p* > |*z*|
Constant	0.3274	0.0794	4.12	0.000*	0.2924	0.0768	3.80	0.000*
Age	0.0154	0.0036	4.34	0.000*	0.0166	0.0034	4.84	0.000*
Agesq	− 0.0001	0.0000	− 3.32	0.001*	− 0.0001	0.0000	− 3.63	0.000*
Married	0.0327	0.0154	2.12	0.034*	0.0197	0.0150	1.32	0.188
Age left school	− 0.0039	0.0015	− 2.66	0.008*	− 0.0037	0.0014	− 2.61	0.009*
11121	0.1621	0.0094	17.19	0.000*	0.1619	0.0094	17.21	0.000*
21211	0.1286	0.0093	13.86	0.000*	0.1285	0.0093	13.89	0.000*
12212	0.0443	0.0105	4.20	0.000*	0.0441	0.0105	4.19	0.000*
13224	− 0.2478	0.0095	− 26.13	0.000*	− 0.2479	0.0095	− 26.22	0.000*
23242	− 0.3081	0.0095	− 32.28	0.000*	− 0.3082	0.0095	− 32.37	0.000*
23314	− 0.2541	0.0105	− 24.15	0.000*	− 0.2545	0.0105	− 24.25	0.000*
ATLQL_stan_					− 0.0693	0.0072	− 9.65	0.000*
	Overall *R*-sq = 0.2695				Overall *R*-sq = 0.3085			

Table 3 also shows the effect of attitudes on TTO values in the last four columns. The term ATLQL_stan_ is significant as expected, and for every 1 standard deviation increase in the attitude scale individuals value states on average 0.0693 lower. Therefore, respondents that express a preference for quality of life over length of life typically trade away more time which results in lower values.

A similar process of elimination was used to derive a base logistic regression model for the choice data that is shown in Table 4. We can see from this that there are differences in the choices for the mild and moderate health states (i.e., moderate, and moderate × years are significant). The negative coefficients indicate that people were less likely to choose the life with more life years and less quality of life when the health states were moderate rather than mild. This suggests that people presented with the moderate set of health states may have been sensitised more towards making choices based on quality of life rather than length of life. Table 4 also shows the effect of attitudes on choice decisions in the last four columns. The term ATLQL_stan_ is not significant as anticipated. The interaction term on ATLQL_stan_ × years_*δ*_ is significant (*p* value 0.000) and negative. Respondents with a higher ATLQL_stan_ score are, therefore, more likely to choose the life with the higher quality and the fewer years. For 1 standard deviation decrease in attitudes, individuals would require an extra 0.038 years (2 weeks) in order for their probability of choosing Life A to remain constant. Sensitivity analysis was run to explore if the exclusion of the two health states in the pooled data set could explain the results, but we found qualitatively similar results when these values were retained. We also ran the model with three-way interactions and found again that we were unable to reject the model that attitudes continued to influence choices (see Online Appendix 4).

**Table 4:** Choices

	Base model				Base model with attitudes			
Latent propensity to choose Life A	Coefficient	Std. err.	*z*	*p* > |*z*|	Coefficient	Std. err.	*z*	*p* > |*z*|
Constant	1.1295	0.1354	8.34	0.000*	1.0567	0.1376	7.68	0.000*
Years	0.0935	0.0167	5.59	0.000*	0.0809	0.0173	4.69	0.000*
Male	− 0.0558	0.0738	− 0.76	0.449	− 0.0550	0.0748	− 0.74	0.462
Age	− 0.0017	0.0027	− 0.62	0.534	− 0.0019	0.0027	− 0.79	0.484
Moderate	− 0.4787	0.0741	− 6.46	0.000*	− 0.5018	0.0752	− 6.67	0.000*
Male × years	0.0213	0.0096	2.20	0.028*	0.0146	0.0099	1.48	0.140
Age × years	0.0008	0.0004	2.27	0.023*	0.0011	0.0003	3.02	0.003*
Moderate × years	− 0.0917	0.0107	− 8.55	0.000*	− 0.0940	0.0110	− 8.55	0.000*
ATLQL_stan_					0.0715	0.0384	1.86	0.063
ATLQL_stan_ × *years*					− 0.0378	0.0055	− 6.84	0.000*

*Significant at the 5% level

## Discussion

In this paper, we explore the extent to which the trade-off valuations fully capture attitudes that are relevant to health-related choices. Our hypothesis is that attitudes towards length and quality of life influence TTO values, but should no longer affect a set of related choices that are based on respondents’ own TTO scores.

As anticipated, we find that TTO values are influenced by respondents’ attitudes towards the length and quality of life, with a 1 Std Dev. increase predicting TTO values of 0.069 lower. This is a change associated with moving from the 50th to 83rd percentile on the attitude scale and so would be thought of as a clinically important difference in other contexts and similar in size to that of background variables like age and gender. Finally, the attitude variable modestly increases the ability to explain the variation in willingness to trade life years. For example, the ability to explain variation in TTO values increased from 0.2695 with the baseline model alone to 0.3085 with baseline model and attitude variables.

We find the somewhat surprising result that though the TTO responses are affected by respondents’ attitudes towards the length and quality of life, attitudes have a residual impact on choices. In particular, the significant interaction between attitudes and years in the choice questions is − 0.0378 and suggests that respondents who preferred quality of life over length of life still preferred the life with the higher quality and the fewer years in the choices. Hence, it seems that using respondents’ own TTO scores to set up choices over two lives which they ought to be indifferent between is picking up some residual attitudinal impact.

Why might there be a residual impact of attitudes in these choices? The first explanation for this result might be around how we set up these choices. Most studies that estimate TTO values for QALY assume a linear QALY model holds (i.e., no discounting) [18–20]. However, for the purposes of this study, we felt the linear QALY model to be a too restrictive form and we instead based the choices on a more flexible model that allowed for discounting of the QALYs over time (based on the assumptions of mutual utility independence and constant proportional trade-off) [15]. While space does not permit a detailed discussion of the method here, we did run a parallel study in which the direct choices were set up in such a way that they relied only on transitivity. The patterns in that data suggest that failures of these assumptions are *not* the main drivers of the results reported here.

Another possible explanation suggests that although the TTO responses reflect attitudes, they are not doing so adequately, so that people are not trading sufficiently in their TTO responses, so the choice questions are still picking up a residual impact of attitudes. This suggests at least here that choices are able to capture additional attitudinal issues better than TTO and raises the question about which is the better method to use. One crucial difference between TTO and the choices presented to respondents here is, of course, that each state is valued against normal health and death in TTO, while two health states are being valued ‘head to head’ in the choices. It is plausible that comparing two states directly focuses attention on differences in quality of life to a greater extent than in the TTO. As most interventions involve moving the patient from one health state to another, and it is ‘moves’ between health states that are commonly valued in economic evaluations, it could be argued that the ‘head to head’ evaluations are most appropriate. Therefore, if value elicitation exercises are to be more focused on policy orientated questions, it would seem the choice questions comparing different periods in ill-health are closer to these policy questions than TTO.

There are a number of limitations of the study. Some of the characteristics which have previously been found to affect TTO values were not included, like number of children, household income and education, so these could have some underlying impact on the responses, but could not explain the continued influence of attitudes in the choice questions. A further limitation of the study design is that health states are not randomised to Life A and Life B in the direct choice, and the strong preference for Life A could, of course, indicate a tendency to favour the left hand option. However, again, this limitation is unlikely to explain the continued influence of attitudes in the choice questions. Finally, we used the TTO method and a limited number of health states so it is possible that our result might be influenced by the elicitation method used and the health states included.

We conclude that although the TTO responses reflect attitudes these attitudes continued to affect health-related choices. Our study suggests that choices are able to capture additional attitudinal issues compared to the TTO and raises the question about which is the better method: a method that values health states against normal health and death as in TTO or a method that compares two health states ‘head to head’ as in choice.

## Electronic supplementary material

Below is the link to the electronic supplementary material.

Supplementary material 1 (DOCX 76 KB)

## Footnotes

1 These variables were added to the model independently of the other and neither were individually significant.
