# Supplementary material for: Do time trade-off values fully capture attitudes that are relevant to health-related choices?
Source: Eur J Health Econ. 2018 Dec 31;20(4):559–68. doi: 10.1007/s10198-018-1017-8 (PMC6517563; doi:10.1007/s10198-018-1017-8)
Supplement: Supplementary file 1 — Supplementary material 1 (DOCX 76 KB) [file 10198_2018_1017_MOESM1_ESM.docx]

**Appendix 1. Exclusions in the dataset**

Initial dataset

**1462**

Excluded due to not

completing questionnaire

**5**

Dataset of size

**1457**

Excluded due to not

providing consent

**11**

Dataset of size

**1446**

Excluded due to not

reading information sheet

**90**

Dataset of size

**1356**

Excluded due to completing

in under 4 minutes

**17**

‘Cleaned’ analysis dataset

**1339**

*Figure A2*

*Initial exclusions made to dataset*

**Appendix 2**

The attitude items were summed using equation 1 (where A_1_ to A_4_ are the attitude statements 1 to 4). The attitude scale was then standardised using equation 2 to create a summary measure of attitudes towards length and quality of life which is referred to as *ATLQL_stan,_* throughout the analyses (where $\sigma$ is standard deviation and $\bar{x}$ is the mean attitude score). The ATLQL score is highest for those who indicated quality of life was most important and lowest for those who considered length more important.


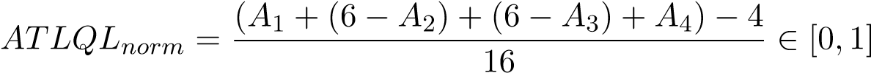
 (1)


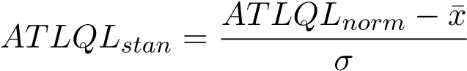
 (2)

**Appendix 3 –Pooling data across the different TTO variants**

We ran a regression to explore the extent to which we could pool responses from the four TTO variants to which respondents were randomised involving slightly different elicitation procedures.

The regression model that was constructed containing dummy variables representing the four TTO variants and all seven states, with a dummy for the severity of the health states to which the respondent was randomised (Mild and Moderate). Also included were interaction terms (cross products) between states and TTO variants. This regression was run to establish if any variants were significantly affecting elicited TTO values. A significant *p*-value for a given variant-term^^[[1]](#footnote-1)^^ would indicate the TTO values generated from this variant were significantly different to those obtained under other variants, and therefore could not be pooled together. In the event that particular variant-terms were found to be significant, it was determined whether the values obtained from these variants could be pooled (and which if any must be dropped) by means of an *F*-test. The regression was run on the cleaned dataset of 1339, and the results are shown in Table 1).

Here we label these variants IS: Iterative Sequential; NIS: Non-iterative Sequential; IC: Iterative Concurrent; NIC: Non-iterative Concurrent.

**Table 1** Model (1) Output for Effect of TTO Variant on TTO Values

| Util | Coefficient | Std. Err. | z | *p*>\|z\| |
| --- | --- | --- | --- | --- |
| 11121 | 0.1632 | 0.0209 | 7.83 | 0.000* |
| 21211 | 0.1387 | 0.0202 | 6.87 | 0.000* |
| 12212 | 0.0788 | 0.0206 | 3.83 | 0.000* |
| 13224 | -0.2540 | 0.0213 | -11.92 | 0.000* |
| 23242 | -0.2972 | 0.0216 | -13.78 | 0.000* |
| 23314 | -0.2454 | 0.0215 | -11.43 | 0.000* |
| Moderate | 0.0149 | 0.0363 | 0.41 | 0.681 |
| IS | 0.0220 | 0.0346 | 0.64 | 0.524 |
| NIS | 0.0230 | 0.0352 | 0.65 | 0.514 |
| IC | -0.0219 | 0.0345 | -0.63 | 0.526 |
| 11121_ IS | -0.0212 | 0.0283 | -0.75 | 0.454 |
| 21211_ IS | -0.0235 | 0.0278 | -0.84 | 0.399 |
| 12212_ IS | -0.0942 | 0.0281 | -3.35 | 0.001* |
| 13224_ IS | 0.0229 | 0.0289 | 0.79 | 0.428 |
| 23242_ IS | 0.0054 | 0.0291 | 0.19 | 0.853 |
| 23314_ IS | -0.0055 | 0.0290 | -0.19 | 0.849 |
| Even_ IS | 0.0209 | 0.0498 | 0.42 | 0.675 |
| 11121_ NIS | 0.0243 | 0.0294 | 0.83 | 0.409 |
| 21211_ NIS | 0.0047 | 0.0285 | 0.17 | 0.868 |
| 12212_ NIS | -0.0281 | 0.0292 | -0.96 | 0.336 |
| 13224_ NIS | -0.0393 | 0.0296 | -1.32 | 0.185 |
| 23242_ NIS | -0.0575 | 0.0299 | -1.92 | 0.055 |
| 23314_ NIS | -0.0657 | 0.0299 | -2.20 | 0.028* |
| Even_ NIS | 0.0262 | 0.0506 | 0.52 | 0.604 |
| 11121_ IC | 0.0095 | 0.0283 | 0.34 | 0.736 |
| 21211_ IC | -0.0052 | 0.0278 | -0.19 | 0.852 |
| 12212_ IC | -0.0469 | 0.0282 | -1.66 | 0.096 |
| 13224_ IC | 0.0206 | 0.0287 | 0.72 | 0.474 |
| 23242_ IC | -0.0105 | 0.0290 | -0.36 | 0.718 |
| 23314_ IC | -0.0055 | 0.0289 | -0.19 | 0.849 |
| Even_ IC | -0.0206 | 0.0496 | -0.42 | 0.677 |
| Constant | 0.6524 | 0.0250 | 26.12 | 0.000* |

*Significant at the 5% level.

This regression used variant NIC and state 13122 as the base case and therefore included no dummies for this variant, state or their interactions.

The dummies for the state being valued are significant as expected. The only other terms which are significantly contributing to the TTO values are 12212×*IS* ( *p*-value = 0.001) and 23314×*NIS* ( *p*-value = 0.028). We interpret that - relative to the base-case variant, IS does cause respondents to give systematically different values for health state 12212, and similarly for 23314 under variant NIS. These results suggest that we cannot pool data collected from the four variants in their entirety.

We test whether dropping the 12212×*IS* and 23314×*NIS* terms would allow us to pool the remaining values across variants and health states. We conduct an *F*-test with Model (1) reported in table 1 as the unrestricted, and Model (2) as the restricted model.

$util=\propto+\beta_{1}11121+\beta_{2}21211\epsilon+\beta_{3}12212+\beta_{4}13224+\beta_{5}23242+\beta_{6}23314+\beta_{7}EVEN+\beta_{8}12212 x IS+\beta_{9}23314 x NIS+\epsilon$ Model (2)

Model (2) contains only two variant-terms, 12212×*IS* and 23314×*NIS*, which were significant in Model (1). The test returns a *p*-value of 0.1191 which is not significant at the 5% level and therefore there is no significant difference between the way the two models fit the data. That is, 12212×*IS* and 23314×*NIS* jointly influence participants’ values to the same extent as 12212×*IS* + 23314×*NIS* + {*All other variant-terms*}. A corollary of this is that if these variables 12212×*IS* and 23314×*NIS* were to be dropped - along with the observations to which they apply^^[[2]](#footnote-2)^^, the remaining values elicited across all four variants could be pooled. Model 1 was rerun on the dataset with the two variant terms omitted, and confirms that no variant-term is now contributing to the values.

**Appendix 4**

We ran the choice regression with three-way interactions and used a chi-test to determine whether the dummies and their interactions were simultaneously zero. This is similar to testing for significant differences between a model with these variant variables added and a model without them, i.e. the difference between full model and reduced models.

**Table 1** Choices

|  | Base Model | | | | Base model with attitudes | | | |
| --- | --- | --- | --- | --- | --- | --- | --- | --- |
| *Latent propensity to choose Life A* | Coefficient | Std. Err. | z | *p*>\|z\| | Coefficient | Std. Err. | z | *p*>\|z\| |
| Constant | 1.1295 | 0.1354 | 8.34 | 0.000* | 1.078 | 0.1376 | 7.68 | 0.000* |
| Years | 0.0935 | 0.0167 | 5.59 | 0.000* | 0.0871 | 0.0170 | 4.92 | 0.000* |
| Male | -0.0558 | 0.0738 | -0.76 | 0.449 | -0.0550 | 0.0748 | -0.74 | 0.462 |
| Age | -0.0017 | 0.0027 | -0.62 | 0.534 | -0.0005 | 0.0027 | -0.19 | 0.853 |
| Moderate | -0.4787 | 0.0741 | -6.46 | 0.000* | -0.5080 | 0.0754 | -6.74 | 0.000* |
| Male$\boldsymbol{\times}$ years | 0.0213 | 0.0096 | 2.20 | 0.028* | 0.0104 | 0.0099 | 1.05 | 0.293 |
| Age$\boldsymbol{\times}$ years | 0.0008 | 0.0004 | 2.27 | 0.023* | 0.0011 | 0.0003 | 3.41 | 0.001* |
| Moderate_$\boldsymbol{\times}$years | -0.0917 | 0.0107 | -8.55 | 0.000* | -0.0947 | 0.0109 | -8.71 | 0.000* |
| *ATLQL_stan_* |  |  |  |  | 0.0476 | 0.0386 | 1.24 | 0.215 |
| *ATLQL_stan_*$\boldsymbol{\times}$*Y ears* |  |  |  |  | -0.0236 | 0.0179 | -1.32 | 0.187 |
| *ATLQL_stan_*$\boldsymbol{\times}$ *Y ears*$\boldsymbol{\times}$*male* |  |  |  |  | 0.0334 | 0.0102 | 3.33 | -0.001* |
| *ATLQL_stan_*$\boldsymbol{\times}$*Y ears*$\boldsymbol{\times}$*age* |  |  |  |  | -0.0027 | 0.0004 | -0.73 | -0.465 |
| *ATLQL_stan_*$\boldsymbol{\times}$ *Y ears*$\boldsymbol{\times}$*Moderate* |  |  |  |  | -0.0335 | 0.0107 | -3.14 | 0.002* |

*Significant at the 5% level.

We find that the chi-squared tests of differences for a model including the dummies and their interactions were statistically significant (e.g. non-iterative sequential chi2(5) = 39.07 Prob > chi2 = 0.000). In this model we found that attitudes continue to affect choices when three- way interactions are included.

1. We will use ‘variant-term’ to refer to any term included in the regression models which contains one of *IC*, *NIC* or *NIS* either as a cross product or alone. [↑](#footnote-ref-1)
2. These numbered 167 and 169 respectively - note however that no respondents were dropped, only one value for each of the IS and NIS respondents [↑](#footnote-ref-2)
